# Aversive learning shapes neuronal orientation tuning in human visual cortex

**DOI:** 10.1038/ncomms8823

**Published:** 2015-07-28

**Authors:** Lisa M. McTeague, L. Forest Gruss, Andreas Keil

**Affiliations:** 1 Department of Psychiatry and Behavioral Sciences, Medical University of South Carolina, Charleston, South Carolina 29425, USA; 2 Department of Psychology, University of Florida, Gainesville, Florida 32611, USA; 3 Center for the Study of Emotion and Attention, University of Florida, Gainesville, Florida 32611, USA

## Abstract

The responses of sensory cortical neurons are shaped by experience. As a result perceptual biases evolve, selectively facilitating the detection and identification of sensory events that are relevant for adaptive behaviour. Here we examine the involvement of human visual cortex in the formation of learned perceptual biases. We use classical aversive conditioning to associate one out of a series of oriented gratings with a noxious sound stimulus. After as few as two grating-sound pairings, visual cortical responses to the sound-paired grating show selective amplification. Furthermore, as learning progresses, responses to the orientations with greatest similarity to the sound-paired grating are increasingly suppressed, suggesting inhibitory interactions between orientation-selective neuronal populations. Changes in cortical connectivity between occipital and fronto-temporal regions mirror the changes in visuo-cortical response amplitudes. These findings suggest that short-term behaviourally driven retuning of human visual cortical neurons involves distal top–down projections as well as local inhibitory interactions.

## Abstract (web summary)

Sensory cortical tuning is shaped by experience to facilitate coding of features that are predictive of behaviourally relevant outcomes. Here the authors demonstrate that rapid behaviourally driven retuning of human visual cortex involves top–down projections as well as local inhibitory interactions.

## Graphical abstract

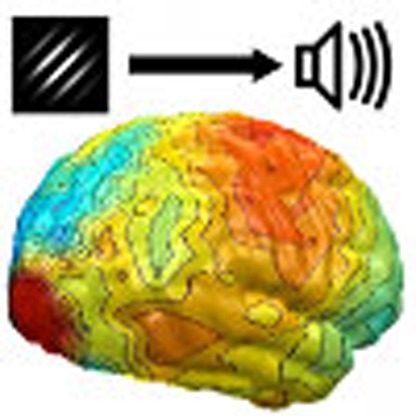

Visual information associated with motivational or affective value typically elicits heightened response amplitudes in visual neurons and is more readily detected and remembered^1^. In the laboratory, fear-conditioning paradigms have been used to study the neural mechanisms underlying such prioritization in both animals^2^ and human participants^3^. This research has suggested that neural populations lower in the visual hierarchy respond more strongly to features signalling threat and danger than to features associated with safety^4^. Paralleling work in the rodent auditory system^5^, these findings have led researchers to hypothesize that the tuning of visual cortical neurons may be altered so that they respond more readily to features that are predictive of behaviourally relevant outcomes^6^. However, the absence of methods for non-invasively quantifying the extent and temporal dynamics of visual cortical changes on a trial-by-trial basis in the human brain has prevented empirical testing of this hypothesis. Here we use single-trial estimates of sustained visual cortical activity evoked by orientation gratings to characterize the time course of neural changes as humans learn to bias perception in favour of a visual feature predicting a noxious, loud noise.

In the visual system, orientation of a contrast stimulus is an obvious choice to study tuning behaviour of neural populations. Orientation selectivity is a crucial property of neurons in retinotopic cortex, which has been studied extensively using animal electrophysiology and psychophysics^7^,^8^. A growing body of work suggests that orientation-tuning functions are not hard-set properties^9^ but are dynamically shaped over time by suppressive mechanisms, such as lateral inhibition^10^ or divisive normalization^11^. Furthermore, orientation selectivity is subject to plastic changes as demonstrated in adaptation paradigms^12^. Importantly, neurons sharing preference for a given orientation are clustered into columns perpendicular to the cortical surface, and arranged such that neighbouring columns overall prefer similar orientations^7^. The present study examined to what extent these structural features are reflected in population activity evoked in human visual cortex by a gradient of orientations in which only one orientation predicts aversive behavioural outcomes. In the human visual system, prioritization of aversive or fear-relevant stimuli has been repeatedly demonstrated among individuals with phobias^13^. Induced or acquired prioritization among healthy participants has also been demonstrated in the laboratory during discriminative classical conditioning^14^, wherein neutral stimuli (that is, the CS+) paired with a noxious event (the unconditioned stimulus (US), for example, a loud noise) elicit facilitated sensory responses, compared with non-paired stimuli (that is, the CS−). If such prioritization leads to sustained amplification in the population activity of neurons sensitive to a specific stimulus feature, then fear conditioning can be utilized to examine lateral interactions among neuronal populations with differential sensitivity to that feature. Specifically, a fear generalization paradigm presenting a range of stimuli varying along a known feature dimension may be well suited to examine inhibitory interactions, which may be obscured if only two stimuli are compared.

Here we examined the steady-state visual evoked potential (ssVEP) elicited in visual neurons by rapidly and regularly phase-reversing Gabor gratings differing in orientation, during differential fear conditioning. The ssVEP provides a direct measure of neural mass activity generated in retinotopic visual areas low in the visual cortical hierarchy^15^. Its amplitude is modulated not only by physical stimulus properties such as brightness and contrast but also by cognitive processes such as selective attention^16^. Previous work has established robust amplification of the ssVEP elicited by CS+ compared with CS− stimuli during classical fear conditioning within a single session^14^,^17^. Because ssVEPs represent ongoing, sustained engagement of visual neuron populations, this rapidly emerging effect has been attributed to re-entrant modulation of visual cortex by anterior areas^18^. This explanation has been corroborated by the fact that extinction learning abolishes CS+ amplification of the ssVEP within few trials^4^. By contrast, studies extending the conditioning regime over hundreds of trials across hours and days have reported lasting effects of conditioning on very early transient event-related potentials, which are unlikely to be modulated by re-entrant projections^6^,^19^. Thus, ssVEPs represent an avenue to characterize the large-scale neurophysiology of initial perceptual bias formation, including potential cortico-cortical interactions that co-occur with local visuo-cortical changes. Leveraging the excellent signal-to-noise ratio of ssVEPs, here we quantify trial-by-trial changes in orientation sensitivity of neural mass activity as observers learn to discriminate a noxious stimulus defined by a specific orientation. Seven gratings were used, differing only in orientation, which increased monotonically in 10° steps. Only one grating (45° orientation) served as the CS+. To the extent that experience shapes the orientation tuning of visual neurons at the population level through lateral inhibitory interactions among neighbouring neuron populations, we expected that fear conditioning would prompt the greatest amplification for the CS+ orientation, and significant reduction for the CS− orientations closest to the CS+. Such a ‘Mexican hat’ tuning pattern is in contrast to the alternative hypothesis of a generalization gradient, in which ssVEP amplitude is expected to gradually decrease with angular distance from the CS+.

The generalization hypothesis is supported by early animal work, particularly spectral generalization in pigeons^20^,^21^. Contemporary work on acquired fear in humans has shown similar gradients, with the magnitude of defensive responses to non-threat cues increasing as a function of their similarity to the CS+. These generalization gradients tend to take the shape of a quadratic function and have been evident across measures of subjective distress, perceived risk, startle reflex responding and cortico-limbic blood oxygenation^22^,^23^. As a mean of bridging this work with visual neuroscience, startle reflex responding and subjective ratings of emotional experience were recorded concurrent with ssVEPs in the present study. Findings support the hypothesis that lateral inhibitory interactions in the visual system coincide with quadratic generalization patterns in efferent defensive reflexes and subjective distress. This pattern of findings is in line with the notion that changing motivational relevance of a stimulus prompts different patterns of adaptive change in sensory systems compared with adaptations in efferent reflex systems.

## Results

### Pooled occipital cortical activity

**Figure 1:**
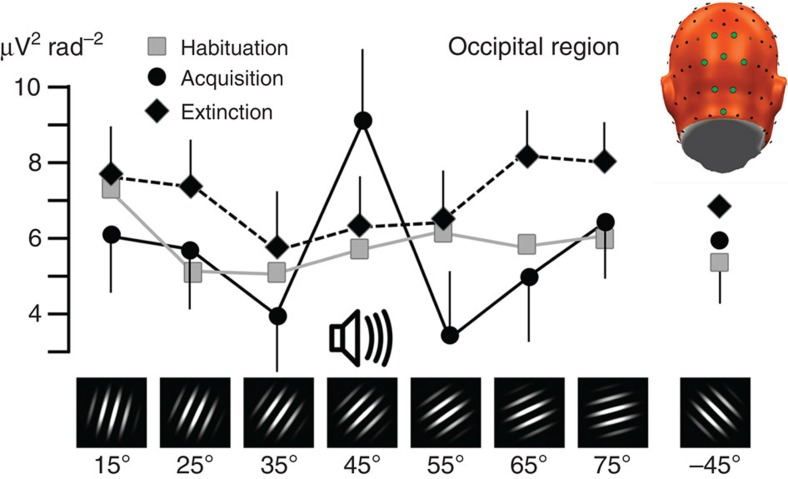
Occipital retuning during aversive conditioning. Changes in grand mean (*N*=15) visual electrocortical activity is shown for each phase of the experiment (habituation, acquisition and extinction) and for each orientation. Regional means of ssVEP amplitudes in CSD (Laplacian) space, averaged across occipital midline sensor locations, were used to estimate the occipital cortex surface potential. The inset shows a back view of the electrode array used, with sensor locations used for averaging highlighted in green. A pronounced Mexican-hat pattern is visible during the acquisition phase, consistent with lateral inhibitory interactions: Amplification of the 45° orientation, which was paired with a noxious sound, is accompanied by relative suppression of proximal orientations (55° and 35°). Error bars (s.e.m.) are shown for the acquisition and extinction phases.

The grating stimuli were presented in a phase-reversing fashion (switched between in-phase and anti-phase images 14–15 times per second), evoking pronounced oscillatory steady-state potentials in visual cortex at the same frequency. The ssVEP signals were converted to current source density (CSD) estimates and the spectral power at the driving (ssVEP) frequency determined. Pooled occipital activity was then obtained by averaging CSD spectral power at the ssVEP frequency across eight sensor locations over the occipital pole (see Fig. 1, top right), for each orientation separately. As expected, mean comparisons with repeated-measures analysis of variance (ANOVA) showed no effect of orientation during the initial habituation phase, *F*(7,98)=0.50, not significant (NS). Accordingly, planned contrast fitting models of lateral inhibition (Mexican hat) and generalization (quadratic) patterns were not significant (*F*s<1.9). By contrast, population activity during the acquisition phase demonstrated clear evidence of modulation by orientation (*F*(7,98)=3.59, *P*<0.01), being robustly modelled as a Mexican-hat pattern (*F*(1,14)=7.22, *P*<0.01), as shown in Fig. 1a. This lateral inhibition pattern in occipital activation subsided during the extinction phase (*F*(1,14)=2.06, NS) in which the ANOVA showed no effect of orientation, *F*(7,98)=1.68, NS.

### Parietal cortical activity

**Figure 2:**
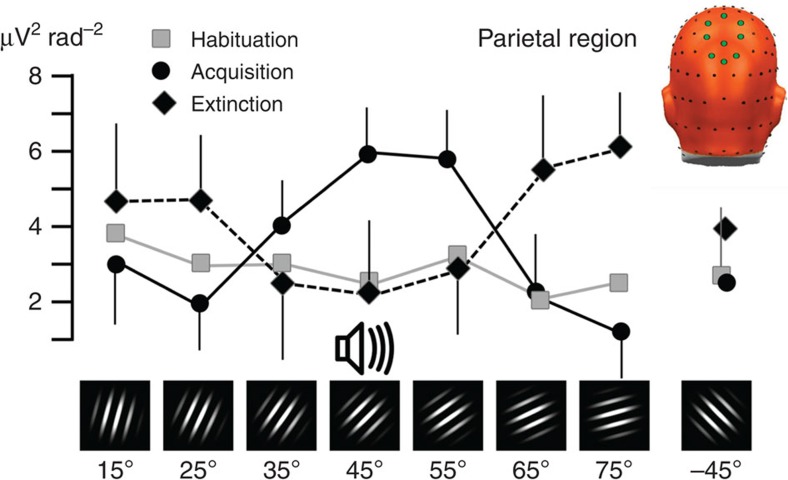
Parietal retuning during aversive conditioning. Grand mean (*N*=15) pooled visual electrocortical activity over parietal cortical sites is shown for each phase of the experiment (habituation, acquisition and extinction) and for each grating orientation. Spectral power of ssVEP current source density is shown, averaged across parietal midline sensor locations, shown as green circles in the top right inset. Note the generalization (quadratic) pattern with amplification of the 45° grating that was paired with noise, and a monotonic decline to distal orientations, specifically during fear acquisition. Error bars (s.e.m.) are shown for the acquisition and extinction phases.

Similar to the sensors over occipital cortex, parietal areas did not show sensitivity to different orientations during the initial habituation phase. During acquisition, the greatest power enhancement was observed for the CS+ orientation (45°) and progressive decrement of ssVEP amplitude was observed with increasing dissimilarity to the CS+, *F*(7,98)=2.96, *P*<0.05. This pattern was best modelled by a quadratic (generalization) contrast (*F*(1,14)=10.41, *P*<0.01; Fig. 2). Interestingly, parietal activation during extinction showed a reversed quadratic (generalization) effect (*F*(1,14)=5.56, *P*<0.05), suggesting an active process counteracting the generalization acquired during the conditioning phase.

### Blink magnitude to acoustic startle probes

**Figure 3:**
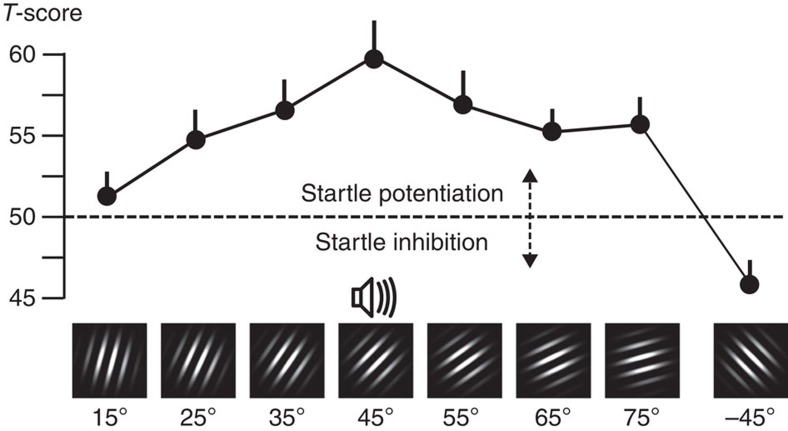
Reflex physiology changes during aversive conditioning. Grand mean (*N*=15) startle reflex magnitude (expressed in standardized *T*-scores) for each orientation, during the acquisition phase of the experiment. Paralleling parietal cortical engagement, a quadratic fear generalization pattern is visible, with amplification of the 45° noise-paired stimulus and monotonic decline to distal orientations. Error bars reflect the s.e.m.

During the acquisition phase, the startle eye blink measures of aversive conditioning showed a pattern similar to the parietal cortical activation during the same experimental phase: the greatest startle potentiation was observed when viewing the CS+ orientation (see Fig. 3), accompanied by a monotonic decrease towards distal orientations. This resulted in a pattern consistent with fear generalization, which was best fit by the quadratic model (*F*(1,14)=17.51, *P*<0.01). Interestingly, reliable inhibition was observed to the −45° cue (Fig. 3, right), even in relation to the mean of the other two cues that were most dissimilar to the CS+ (that is, 15, 75) (*F*(1,14)=16.17, *P*<0.01). This suggests a robust attentional suppression of the defensive startle reflex, possibly owing to perceptual dissimilarity or to the related perception of safety. Evidence of modestly persisting generalized defensive engagement also emerged during extinction in startle reactivity, with the generalization model exhibiting a significant fit (*F*(1,14)=6.19, *P*<0.05).

### Subjective experience

**Figure 4:**
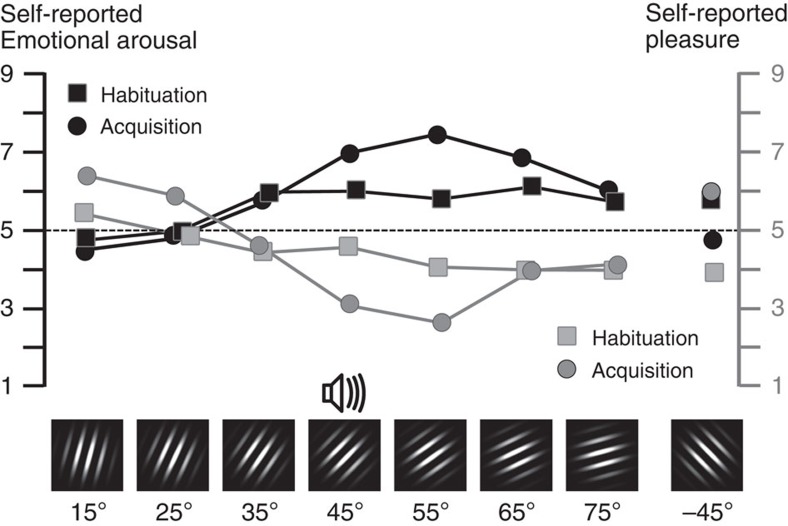
Self-report changes during aversive conditioning. Grand mean (*N*=15) self-reported emotional arousal (black lines, left scale) and pleasure (grey lines, right scale), comparing post-habituation (squares) and post-acquisition ratings (circles) for each orientation, on the SAM nine-point scale.

From post-habituation to post-acquisition, the verbal reports on the nine-point Self-Assessment Manikin (SAM) scale also showed a shift to patterns indicative of generalized distress as evidenced in endorsed displeasure (*F*(7,98)=7.11, *P*<0.01), best modelled by a generalization (quadratic) pattern during acquisition (*F*(1,14)=18.29, *P*<0.001), as shown in Fig. 4. The same pattern was observed when considering reports of emotional arousal (*F*(1,14)=26.68, *P*<0.001) and dominance (*F*(1,14)= 5.35, *P*<0.05). Interestingly, subjective responses showed less specificity to the CS+ cue relative to the clear augmentation of responding in startle reflex and parietal occipital activation. Although the generalization pattern was pronounced, ratings of aversion, arousal and lack of control/dominance were similarly extreme for the CS+ cue as well as the 55° grating. Subjective ratings post extinction suggested moderately sustained aversion (generalization contrast: *F*(1,14)=8.72, *P*<0.005), and arousal (generalization contrast: *F*(1,14)=7.35, *P*<0.05), for the former CS+ cue and the most similar gratings. Simultaneously, the generalized lack of control during acquisition subsided during extinction, leading to absence of the generalization contrast (*F*(1,14)=1.71, NS).

### Whole-brain topographies

**Figure 5:**
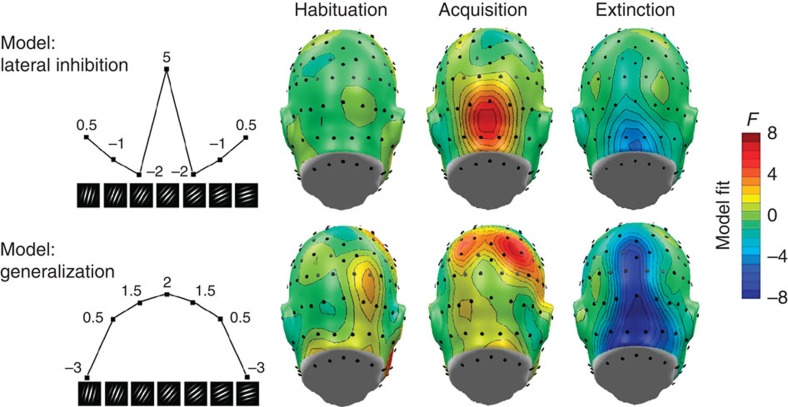
Cortical regions sensitive to aversive learning. Topographical distribution (back views are shown) of planned contrast models testing the competing hypotheses of lateral inhibition (top, Mexican-hat contrast) versus fear generalization (bottom, quadratic contrast) of electrocortical responses across orientations, during the three experimental phases. Weights used for the planned contrasts are displayed for each orientation (left panel). *F*-values exceeding ±4.27 indicate reliable model fits at a given sensor location. Fits that matched the opposite pattern are shown in blue, indicating negative fit. Note the focal lateral inhibition (Mexican hat) pattern evident during acquisition over occipital areas, accompanied by parietal generalization indicated by a quadratic contrast. The tendency to reverse amplification of the noise-paired cue (that is, the 45° orientation) is reflected in the inverted patterns seen during the extinction phase, across parietal and occipital sites.

In a next step, we examined the topographical distribution of learning-related effects by performing planned contrast analyses reflective of the competing hypotheses (Fig. 5, left) at each individual sensor, resulting in topographical maps of fit coefficients (*F*-values) chosen to indicate the strength of the lateral inhibition versus generalization patterns across orientations, during habituation, acquisition and extinction. Visual cortical activation corresponding to the lateral inhibition (Mexican hat) pattern emerged during acquisition and more focally during extinction (Fig. 5, top). By contrast, pronounced quadratic amplitude modulation consistent with a generalization gradient emerged at parietal–occipital sites during acquisition. Interestingly, a robust inversion of this generalization pattern was observed for the extinction phase (Fig. 5, bottom), in broad parietal and occipital regions.

### Temporal evolution of learning in visual cortex

**Figure 6:**
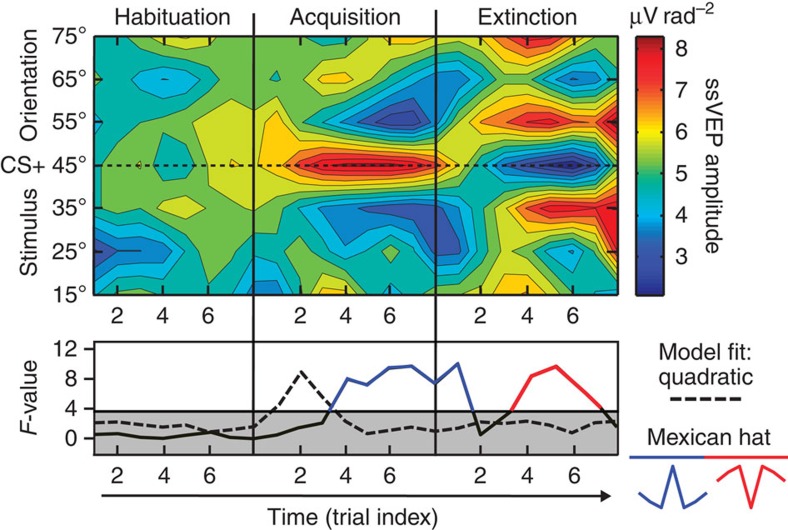
Temporal evolution of orientation tuning during aversive learning. Top: color-coded single-trial amplitude of the visual electrocortical response, across the phases of the experiment. Learning dynamics in visual cortex are shown over eight trials per phase for seven neighbouring orientations, centred around the 45° orientation (the threat cue), which was paired with the noxious sound. Bottom: model fits (planned contrasts) for the competing hypotheses of fear generalization (top) and lateral inhibition (bottom, Mexican hat), calculated for each trial. Note that initial amplification of the threat cue (45°) is followed by increasing relative amplitude reduction for the proximal orientations. Complete reversal of these changes is visible during fear extinction, resulting in fitting an inverse Mexican-hat model, indicated in the lower panel by *F*-values shown in red.

Averaging across all trials in an experimental phase may obscure pertinent dynamics of fear acquisition and extinction effects in visual cortex. Therefore, ssVEP amplitude estimates for single trials were obtained for the locations showing modulation in Fig. 5 around the extended occipital pole, to quantify the evolution of changes in visual cortex for each orientation as learning progressed (see Fig. 6). Critical *F*-values for generalization (quadratic) and lateral inhibition (Mexican hat) patterns in the data were determined by calculating permutation distributions^24^ on data shuffled across orientations within each observer (5,000 permutations), leading to a critical *F*-value of 3.76. Significant selective amplification of the CS+ grating occurred after only two CS+/US pairings, resulting in a significant quadratic trend test (dashed line in Fig. 6, bottom panel). After four pairings, inhibition (suppression) of the orientations proximal to the CS+ orientation is visible, reflected in a significant fit of a Mexican-hat (lateral suppression) pattern. During extinction, these effects reversed after three CS+/US pairings, leading to suppression of the CS+ and amplification of the proximal orientations. This resulted in a reversed inhibition pattern that was statistically significant until the seventh CS+/US pairing.

### Connectivity analyses

**Figure 7:**
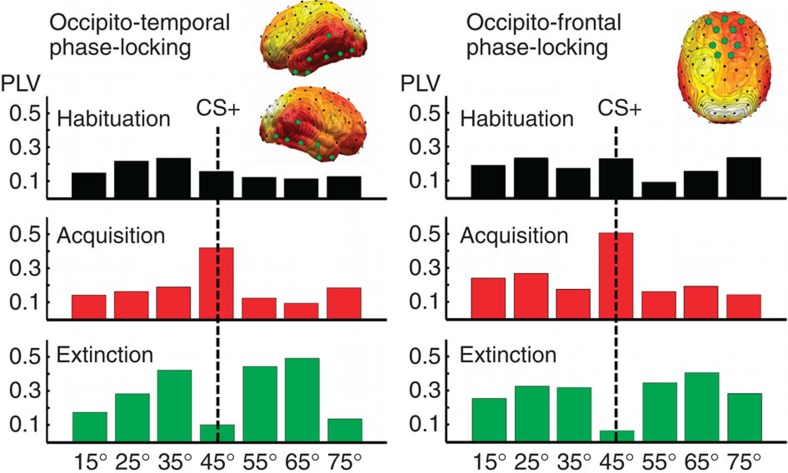
Connectivity changes during visual retuning. Pooled cortico-cortical connectivity relative to a reference site at the occipital pole across the three learning phases, shown for each orientation and for two regions of interest. Both occipitotemporal (left panel, pooled sensor locations shown as green circles) and occipitofrontal (right panel) connectivity show amplification of the CS+ orientation (45°) during acquisition and its suppression during extinction. PLV, phase-locking value.

Inter-site phase locking between a seed location at the occipital pole (electrode site Oz of the international 10–20 system) and the remainder of the sensor array was used as a measure of connectivity and was calculated for each experimental phase and condition (see Fig. 7). The phase-locking value used here^25^ measures the stability of the phase difference between two signals across trials. The phase-locking value is a statistical quantity and we again used permutation distributions on randomly shuffled data to control for multiple comparisons when determining statistical significance.

Across conditions, two regions of significant connectivity with the occipital pole emerged (see top right panels in Fig. 7): a region covering occipitotemporal areas and a region covering medial frontal regions. Phase synchrony between the occipital pole and these two regions varied with the experimental manipulations, paralleling the findings for occipital amplitude. Only the Mexican hat contrast model showed a significant fit to the data, during acquisition (*F*(1,14)= 4.77, *P*<0.05) and extinction (*F*(1,14)=5.96, *P*<0.06), in which an inverted Mexican-hat model again best explained the data.

## Discussion

It has long been established that experience changes sensory systems^5^,^26^. The present study characterized the changes in the orientation selectivity of neural populations while human observers learn that a specific orientation predicts a noxious outcome. Startle reflex responses and subjective ratings of aversion, arousal and control demonstrated that aversive conditioning resulted in defensive mobilization in both physiological reflex and evaluative systems to the CS+ and diminished stepwise with the increasing angular distance of each CS−. Furthermore, the conditioned fear generalization reflected in these measures was modestly sustained throughout extinction. In stark contrast, visual cortical activation showed that amplification of the CS+ orientation following fear conditioning is accompanied by reduction of neural mass activity in response to the most proximal, but not distal orientations. This pattern of results is consistent with a growing body of research in experimental animals suggesting inhibitory interactions among orientation sensitive cells in mammalian visual cortex, and potentially at earlier levels of the visual processing stream such as the thalamic level^27^,^28^. It is important to note, however, that neural mass activity measured using scalp electroencephalogram (EEG) cannot distinguish between two potential mechanisms producing the observed changes: Individual neurons may change their tuning behaviour during aversive learning, but it is also conceivable that the orientation specificity of the ensemble activity changes at the population level. Work in an animal model of sensory fear generalization is needed to address this question.

The fact that experience changes the tuning behaviour of neural mass activity is consistent with reports of sharpened orientation-specific hemodynamic brain activation as a consequence of contingency learning, measured by multi-voxel pattern analysis^29^. In addition, the findings support the notion that passive exposure to contingencies may induce strong changes in orientation sensitivity, potentially mediated through short-term plastic processes^9^ or through recurrent interactions among cortical columns or areas^30^. Specific amplitude enhancement for the CS+ orientation, accompanied by relative suppression of similar orientations may also shed light on previous work in humans using event-related brain potentials: Mixed results of brain potential enhancement versus reduction have been reported in these studies that often used complex conditioned stimuli such as words or complex geometrical figures^31^,^32^. Given the present data, one would predict that only discriminative features of the CS+ will be amplified while other aspects, especially those shared with the CS− will be suppressed. In studies with multi-feature conditioned stimuli, what is being amplified and suppressed may often be difficult to distinguish, and the net result of amplification and reduction may vary. In many cases, CS+ and CS− may share more features than distinguishes them, further complicating the interpretation regarding processes underlying their discrimination.

Amplification of the CS+ orientation and reduction of the proximal CS− orientations were present throughout the acquisition phase. However, visual population activity was also sensitive to extinction, showing a pronounced inversion of the pattern seen during acquisition. Thus, paralleling extinction learning in fear circuits centred around the amygdala^33^, extinction learning in visual cortex involves an active process, in which the CS+ features are suppressed. Assuming inhibitory interactions between proximal orientations, CS+ suppression would lead to heightened response amplitudes for proximal CS− stimuli during extinction—the pattern observed in the present study.

An added benefit of multi-electrode ssVEP recordings is in the availability of topographical information, exploited here by means of the CSD representation of the signal. Studies performing source estimation of the ssVEP have suggested that the ssVEP signal originates to a large extent in retinotopic visual cortex with additional contributions from anterior areas such as V5 (refs 15, 34). It is important, however, to note that observing evidence for primary current sources in primary cortical areas does not exclude the possibility of co-active sources elsewhere in the brain. The present source density patterns show a strong generalization gradient at parietal locations independent of the lateral inhibition (Mexican hat) pattern observed occipitally. This may be taken to indicate that parietal, but not occipital cortical, areas show heightened engagement for orientations similar to the CS+, rather than inhibition of proximal orientations. In addition to mirroring the reflex, autonomic and subjective measures in this and other studies^23^, parietal but not occipital blood oxygen level dependent (BOLD) activation has shown conditioned fear generalization in a very similar conditioning procedure with 10 Hz flickering checkerboard rings^22^. Furthermore, fear generalization in parietal cortical areas is consistent with predictions based on a selective-attention perspective, where similarity with a relevant stimulus results in heightened electrocortical facilitation^35^. Although intuitively appealing, corroboration and replication of these spatial dissociations is needed using measures with greater spatial specificity.

Connectivity analyses conducted on the present data identified widespread areas in temporal and frontal cortex, which showed heightened synchrony with the ssVEP oscillation measured at occipital sensor locations. Across experimental phases and orientations, connectivity between the occipital pole and these anterior regions mirrored the effects observed for the local visuo-cortical ssVEP response amplitude discussed above. Although these analyses do not provide causal or directional links, they are consistent with the hypothesis that short-term changes in sensory sensitivity during fear conditioning may be driven by feedback signals from areas outside visual cortex^18^. Future work using neuroimaging methods with greater spatial resolution may address this question.

A reliable inverse generalization pattern emerged during extinction over occipital and parietal locations. Because this effect was accompanied by the elevation of the orientations distal to the CS+ and amplitude reduction of the CS+ relative to acquisition, it is likely to index an active change of orientation tuning, rather than the mere absence of amplification of the CS+ orientation. Active processes capable of inducing changes in tuning behaviour include adaption^9^, attention^12^ and perceptual learning^36^. The latter process in particular has been extensively studied using, among other tasks, orientation detection paradigms. Theoretical accounts of perceptual learning such as the Reverse Hierarchy Theory^37^ have postulated a neurophysiological sequence underlying the improved performance seen after perceptual learning: The pre-learning naive state is characterized by strong top–down processing in higher-order cortical areas with large receptive fields. Extensive training, however, prompts increased involvement of sensory processes low in the classical visual hierarchy. In the context of the present research, this would suggest that lasting changes in sensory neurophysiology—as opposed to the rapid and reversible changes observed here—would be expected only after extensive aversive conditioning. There is indeed electrophysiological evidence in humans for such a notion^6^,^19^. Future research may employ event-related potentials to quantify the extent to which such changes rely on bidirectional cortical communication or reflect local plastic changes.

Suppression of the CS+ orientation during extinction is not consistent with mere recovery from adaptation back to a baseline state^38^, because such a mechanism would be expected to prompt relative enhancement of the proximal orientations, previously suppressed during the acquisition phase^39^. In a similar vein, enhancement of perceptual sensitivity^40^ and neural population response^34^ in sustained attention tasks has been shown to result in relative suppression for the attended stimulus later in time, suggesting faster adaptation when neural gain is increased^40^, an interpretation that is supported by the present results during extinction. Together, this supports an active learning process during extinction, rather than ‘passive’ fading of the learned brain response. Finally, the inverted generalization pattern in parietal–occipital cortex and the attenuated but persistent enhancement of the CS+ and most similar CS− cues throughout extinction in reflex and subjective measures underscore the expected dissociation between sensory cortical and autonomic/behavioural processes. Inhibitory interactions of visual neurons during aversive learning render adaptive value by maximizing sensory specificity. By contrast, generalization in behavioural and autonomic systems maximizes the organism’s readiness to respond to danger. The outcome of these responses in turn will shape perceptual biases, which thus may be regarded as a determinant as well as a result of adaptive behaviour.

## Methods

### Participants

Fifteen participants (mean=19.2 years; s.d.=1.9; seven female) were recruited through the introductory psychology course at the University of Florida or through flyers and advertisements. All participants scored within the normative range of the Spielberger^41^ State-Trait Anxiety Scale (trait score: mean=32.2; s.d.=9.75) and reported no personal or family history of seizures or photic epilepsy, and had normal or corrected vision. The research was approved by the University of Florida Institutional Review Board, and all participants gave written informed consent before the experimental session. Sample size was determined based on effect sizes seen in previous work with ssVEPs during classical conditioning^14^,^17^.

### Materials and stimuli

High-contrast sinusoidal grating stimuli (maximum Michelson contrast: 95%), filtered with a Gaussian envelope (that is, Gabor patches) and shown on a dark grey background, were the primary stimuli used in the current experiment. The luminance of the dark grey background alone was 1.4 cd m^−2^, and the ambient light level (defined here as the mean luminance of the wall facing the participant) was 0.4 cd m^−2^, as measured with a Gossen MavoSpot 2 luminance meter and cross-validated with a Cambridge Research Systems spectroCAL spectroradiometer. Viewed from a distance of 150 cm, all gratings spanned a visual angle of 5.7° vertically and horizontally. Eight different Gabor orientations were created in Psychtoolbox^42^ running on MATLAB. A grating with 45° orientation (relative to a vertical 0° grating, see Fig. 1, bottom) became the CS+ condition during the acquisition phase. Additional orientations of 15°, 25°, 35°, 55°, 65° and 75° served as CS− conditions and a −45° orientation as an additional control condition. To elicit ssVEPs, two phase-reversed versions of each Gabor patch were rapidly alternated during a given trial, at either 14 Hz (seven observers) or 15 Hz (eight observers). White noise, filtered between 1 and 3,000 Hz, was used for both the startle probe (duration 50 ms) and the unconditioned stimulus (1,000 ms), delivered over headphones at 98 dB sound pressure level.

### Design and procedure

On providing informed consent in the laboratory, participants were seated 150 cm from a SONY 27-inch cathode ray tube monitor, in a sound-attenuated, dimly lit room and the EEG and physiology sensors were attached. Participants were informed that over the course of three phases, a series of flickering shapes would be displayed accompanied by brief sounds. Participants were reminded to remain as still as possible throughout the experiment, and to comfortably maintain gaze on the centre of the screen. No further task was given during EEG recording. At the outset of the experimental procedure (pre-habituation) and then on completion of each phase (habituation, acquisition and extinction), participants were asked to rate each of the Gabor gratings with the SAM^43^ to assess changes in three dimensions of subjective experience: pleasure, emotional arousal and dominance. The SAM is a nine-point self-report scale with graphical anchors (manikins) for different levels of pleasure, emotional arousal and dominance.

The first phase (habituation) consisted of eight pseudorandom presentations of each of the eight gratings (64 trials) with an inter-trial interval varying randomly between 4.5 and 6.5 s, with values drawn from a rectangular function. Subsequently, participants underwent an explicitly instructed acquisition procedure with 100% reinforcement of the CS+, intermixed with presentation of the other Gabor gratings. Before the start of acquisition, participants were presented with the CS+ cue and instructed that it would be accompanied by a loud-noise blast. The acquisition phase was similar to habituation except for a different pseudorandom order of the gratings and on reinforced trials, the CS+ grating was maintained for an additional second until co-termination with the 1-s US noise. For the extinction phase, participants were explicitly informed that for the final series (another pseudorandom series of 64 trials, 8 presentations per grating) the loud-noise blast would no longer be delivered. Acoustic startle probes (50 ms at 98 dB) were administered on 50% of the Gabor presentations (that is, four trials per grating). Trial length was 5,000 ms (70 pattern reversals at 71.43 ms each) for those participants in the 14-Hz group and 4,667 ms (70 pattern reversals at 66.67 ms each) for the 15-Hz group. After completion of the experiment (∼30 min), participants were debriefed. All procedures were approved by the Institutional Review Boards of the University of Florida.

### Data collection/processing

EEG was continuously recorded from a 129-channel sensor net, manufactured by Electrical Geodesics, Inc. EEG was digitized at 250 Hz, with Cz as the recording reference, and band-pass filtered online between 0.1 and 100 Hz. Impedances were kept below 50 kΩ, as recommended by the manufacturer. Subsequently, processing of the recorded data occurred offline, applying a Butterworth low-pass filter with a cutoff (3-dB point) of 40 Hz and 45-dB attenuation at 50 Hz, and converting the data to an average reference. Artifact rejection was implemented through EMEGS software^44^, which uses statistical parameters such as the distribution of the mean, s.d. and gradient of the voltage amplitude to identify outlying channels or trials as well as trial–channel pairs. Sensors contaminated with said artifacts were then interpolated using a statistically weighted, spherical spline interpolation from the full channel set. After artifact correction, an average of 5.8 ssVEP trials per condition (range: 5–8) were retained for analysis, with no significant differences between phases or conditions (all *F*s<1). Trials of the same experimental phase and condition were then averaged in the time domain, forming representations of evoked activity. In addition, artifact-free trials were stored and used for assessment of trial-by-trial changes in orientation selectivity on the population level. To heighten the spatial specificity of the scalp-recorded signal, and to facilitate comparison with previous work on visual aversive conditioning with ssVEPs, the scalp CSD^45^ transform was applied to the averaged scalp voltage data as well as single-trial data. Thus, all voltage time series were converted into CSD time series with the same dimensions (sensor locations by time points) as the original voltage data. The CSD approach is a mapping technique that estimates the potential distribution at the cortical surface. Calculating the CSD does not involve current source estimation by model fitting, but is based on the spatial Laplacian (the second spatial derivative) of the scalp potential. Compared with source-estimation algorithms, the CSD has a unique mathematical solution that does not depend on a priori assumptions or constraints. This procedure mitigates effects of volume conduction and reference dependency of the EEG voltage signal. It thus also assists in reducing spurious connectivity findings. Here the CSD approach described by Junghöfer *et al.*^45^ was used, which is based on spherical spline interpolation and well suited for dense-array EEG montages. The resulting CSD values are represented on a sphere that approximates a cortical surface. All analyses were performed on CSD data, and CSD data (estimates of cortical surface potentials) are shown throughout in figures. For clarity of illustrations, we used a simplified cortical surface for graphing of complex CSD-based distributions such as connectivity data (Fig. 7), and head models (back views) were used to show CSD amplitude differences over the posterior cortical surface.

### Peripheral physiology data collection and reduction

Acquisition of the eye blink component of the acoustic startle reflex response was accomplished using a PC-compatible computer-running VPM software^46^. Electromyography activity over the orbicularis oculi muscle of the left eye was recorded. The raw signal was amplified (5,000) and frequencies below 28 Hz and above 500 Hz were filtered, using a Coulbourn S75-48 bioamplifier. The raw signal was rectified and integrated using a Coulbourn S76-23A contour following integrator, with an actual time constant of 20 ms. To capture startle reflex responses, ongoing activity in the orbicularis oculi muscle was sampled at 20 Hz and was increased to 1,000 Hz for 100 ms before and 250 ms following acoustic startle probe onset. Startle blinks from orbicularis oculi electromyography were reduced offline by a programme that scored each trial for magnitude and onset latency, using an algorithm developed by Globisch *et al.*^47^.

### SsVEP: spectral analysis

**Figure 8:**
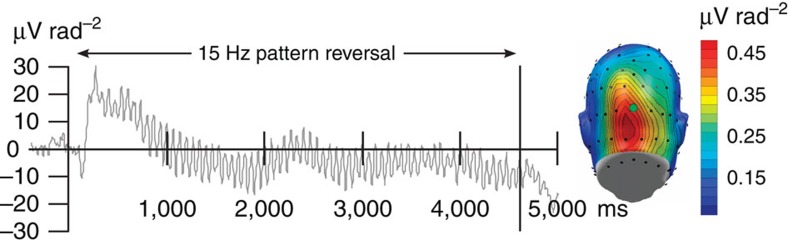
Steady-state evoked potential time series. Left panel: representative ssVEP time series evoked by the pattern-reversing grating stimuli used in this study, shown across all habituation trials for the CS+ orientation (45°), averaged across observers in the 15-Hz group (*N*=8). The right panel shows the topographical distribution of the spectral power of the ssVEP source density, with electrode location Oz used as a reference point for spatial pooling, highlighted by a green circle.

Time domain averages of CSD values at each sensor location for each phase and condition were transformed into the frequency domain using a Fourier transform of the last 4,200 ms (800 sample points) of Gabor grating presentation (before the US presentation in CS+ acquisition trials): In both the 15- and 14-Hz conditions, time domain data were windowed with a short cosine-square window (20 points rise/fall) and then submitted to a discrete Fourier transform (DFT) implemented in MATLAB, resulting in a spectral representation with 0.238 Hz frequency resolution. The segment was selected to eliminate any effects of the brain response to the onset of the flickering train, and was based on prior work indicating that this window would be sensitive to enhanced attention to CS+ cues. More specifically, ssVEP amplitude during CS+ presentation progressively increases as the temporal distance to the US decreases^14^,^48^. Fourier coefficients were normalized by the number of time points and the ssVEP amplitude extracted as the absolute value of the normalized Fourier coefficients at the respective driving frequency (14 and 15 Hz). For statistical analyses, the resulting amplitude estimates were pooled across the Electrical Geodesics, Inc (EGI) sensor corresponding to site Oz of the International 10–20 System, where the spectral amplitude was maximal (see Fig. 8), and its seven nearest neighbours.

Guided by prior findings of an acquired fear generalization gradient in parietal cortex^22^, an additional analysis was conducted at parietal sites, using site Pz and its eight nearest neighbours. Thus, an ssVEP amplitude estimate was generated for each participant, phase and condition, resulting in 24 estimates per participant. To reduce the known large inter-individual variability in ssVEP magnitude, a z-transformation was applied to these 24 estimates, using each individual’s overall mean (across phases and conditions) and s.d.

### SsVEP: single-trial and connectivity analyses

Trial-by-trial estimates of ssVEP amplitude were calculated by means of moving-window averaging across the duration of an ssVEP single trial as described in ref. 49. To avoid contamination with the initial event-related brain potential (ERP) and ensure sensitivity to conditioning effects occurring typically later in the epoch^14^,^48^, the first 800 ms of each epoch were discarded. Subsequently, a moving average window containing four cycles of ssVEP of the respective driving frequency (14 and 15 Hz) was shifted across each remaining segment (the pattern reversal epoch minus the first 800 ms) in steps of one ssVEP cycle. Thus, a total of 55 moving averages were obtained in the 15-Hz group, with the first window starting at 800 ms and the last window at 4,400 ms. In the 14-Hz group, 55 moving averages were likewise obtained, with the last window starting at 4,657 ms. The CSD values within the shifting windows were averaged, resulting in segments containing four cycles of the these averages, which were then transformed into the frequency domain using DFT: after de-trending of each segment and tapering with a cosine-square window (five sample points rise/fall), real and imaginary parts of the 15-Hz and 14-Hz components were obtained. The Fourier coefficients were normalized by the length of the segment and their complex conjugate was calculated as a measure of stimulus-locked ssVEP activity in each trial.

To estimate connectivity between visual cortex and the remainder of the cortical surface covered by the electrode montage, the normalized complex phase was extracted for each segment and trial by dividing the Fourier coefficients at the ssVEP frequency by the power in each segment. The difference of these complex values between each sensor location and electrode site Oz was then averaged across segments and epochs, resulting in a measure of inter-site phase synchrony between the occipital pole and the remainder of the recording array^25^. On the basis of previous work^14^, bilateral frontal areas as well as temporal locations were considered areas of interest for comparing synchrony values across experimental conditions.

### Statistical analyses

The mean normalized signal change at the driving ssVEP frequency for separate occipital and parietal clusters as well as startle reflex responding and subjective aversion, arousal and dominance were submitted to repeated-measures ANOVA, with phase (habituation/acquisition) and orientation as within-subjects factors (15, 25, 35, 45, 55, 65, 75 and −45). Greenhouse Geisser correction addressed sphericity issues. Given the exploratory nature of extinction learning on orientation tuning, the extinction phase was not included in the omnibus ANOVAs; separate repeated-measures ANOVAs were performed for each measure with orientation as the within-subjects factor and follow-up discrimination or generalization contrasts for occipital activation and all other measures, respectively.

To specifically test the competing hypotheses of generalization versus lateral inhibition for the different measures available in this study, we fitted signed *F*-contrast weights representing the mean differences expected under the two competing hypotheses: generalization was modelled as a quadratic trend (weights: −3, 0.5, 1.5, 2, 1.5, 0.5 and −3) across the ordered means (15°–75°) and lateral inhibition was modelled as difference of Gaussians (Mexican hat; weights: 0.5, −1, −2, 5, −2, −1, 0.5; see Fig. 5, left). Multiple comparisons were addressed by calculating permutation distributions for surrogate data in which conditions (orientations and phases) were shuffled within participants, with 5,000 shuffled *F*-tests entering each permutation distribution. The 0.025 and 0.975 tails of these distributions served as the significance thresholds for each dependent variable.

## Additional information

**How to cite this article:** McTeague, L. M. *et al.* Aversive learning shapes neuronal orientation tuning in human visual cortex. *Nat. Commun.* 6:7823 doi: 10.1038/ncomms8823 (2015).
